# Halomonas sp. BS4, A biosurfactant producing halophilic bacterium isolated from solar salt works in India and their biomedical importance

**DOI:** 10.1186/2193-1801-2-149

**Published:** 2013-04-10

**Authors:** Mariathason Birdilla Selva Donio, Fernando Arul Ronica, Vijayaragavan Thanga Viji, Subramanian Velmurugan, John Selesteen Charles Adlin Jenifer, Mariavincent Michaelbabu, Prasenjit Dhar, Thavasimuthu Citarasu

**Affiliations:** Centre for Marine Science and Technology, Manonmaniam Sundaranar University, Rajakkamangalam, Kanyakumari Dist, Tamilnadu 629502 India; Department of Veterinary Microbiology, COVAS, CSKHPKV, Palampur, Himachal Pradesh 176062 India

**Keywords:** Biosurfactants, Halomonas, White spot syndrome virus (WSSV), Antimicrobial activity

## Abstract

Halophilic bacteria were isolated from Thamaraikulam solar salt works in India. After routine biosurfactant screening by various methods, the biosurfactant producing bacteria, Halomonas sp BS4 was confirmed by 16 S rRNA sequencing. The growth optimization of Halomonas sp BS4 revealed their optimum growth at 8% NaCl and 6-8 pH in the growth medium. Further the partially purified biosurfactants were characterized by TLC, FTIR and GC-MS analysis. GC-MS results revealed that, the partial purified biosurfactants contain 1, 2-Ethanediamine N, N, N’, N’-tetra, 8-Methyl-6-nonenamide, (*Z*)-9-octadecenamide and a fatty acid derivative. Pharmacological screening of antibacterial, antifungal, antiviral and anticancer assays revealed that, the biosurfactant extracted from Halomonas sp BS4 effectively controlled the human pathogenic bacteria and fungi an aquaculturally important virus, WSSV. The biosurfactant also suppressed the proliferation of mammary epithelial carcinoma cell by 46.77% at 2.5 μg concentration. Based on these findings, the present study concluded that, there is a possibility to develop eco-friendly antimicrobial and anticancer drugs from the extremophilic origin.

**Electronic supplementary material:**

The online version of this article (doi:10.1186/2193-1801-2-149) contains supplementary material, which is available to authorized users.

## Introduction

Molecular activities individually and in mixtures are initials and signatures for originating scientific simulations and frameworks for academic as well as new industrial upcoming. It is more important with biomolecules such as egg-phosphatidylcholine (EPC) which being weakly polar are involved in molecular interactions as emulsifying agent (Ponder and Case, 2003; Warshel et al. 2006). Surfactants and emulsifiers are indispensable components of daily life (Siegmund, 2002). Microbial compounds that exhibit pronounced surface and emulsifying activities are classified as biosurfactants. Biosurfactants are biological surface-active compounds released by microorganisms that can have some influence on interfaces (Yeh et al. 2006; Joshi et al. 2008). Biosurfactants are amphiphilic compounds produced on living surfaces, mostly microbial cell surfaces or excreted extracellularly and contain hydrophobic and hydrophilic moieties. They reduce surface tension and interfacial tension between individual molecules at the surface and interface respectively (Karanth et al. 1999).

Recently, an increase in the concern about environmental protection has caused the development of cost-effective bioprocesses for biosurfactant production (Morita et al. 2007). The microbial surfactants (MS) are complex molecules covering a wide range of chemical types including peptides, fatty acids, phospholipids, glycolipids, antibiotics, lipopeptides, etc. Research in the area of biosurfactants has expanded quite a lot in recent years due to its potential use in different areas. Most of the biosurfactants used as antibacterial, antifungal or antiviral agents are required in very low concentrations as expressed by their MIC (minimum inhibitory concentration) index. This factor makes biosurfactants highly sought after biomolecules for present and future applications as fine specialty chemicals, biological control agents, and new generation molecules for pharmaceutical, cosmetic and health care industries. Biosurfactants are not only useful as antibacterial, antifungal and antiviral agents; they also have potential for use as major immunomodulatory molecules, adhesive agents and even have use in vaccines and gene therapy. Biosurfactants have several advantages compared with synthetic surfactants: lower toxicity, higher biodegradability, better environmental compatibility, higher foaming, higher selectively and specific activity at extreme temperatures, pH and salinity, and the ability to be synthesized from renewable feedstock (Kumar et al. 2006).

The involvement of biosurfactants in microbial adhesion and detachment from surfaces has been previously investigated. A surfactant released by *Streptococcus thermophilus* has been used for fouling control of heat-exchanger plates in pasteurizers as it retards the colonization of other thermophilic strains of Streptococcus responsible for fouling (Busscher et al. 1996). Possible applications of biosurfactants as emulsifying agents for drug transport to the infection site, as agents supplementing the pulmonary surfactant and as adjuvants for vaccines were suggested by Kosaric (Kosaric, 1996). In the present study, we have studied the production, optimization, characterization and biomedical application of biosurfactants obtained from halobacterium, Halomonas sp BS4 isolated from solar salt works.

## Materials and methods

### Isolation and characterization of Halomonas sp BS4

Condenser water having a salinity of 155% was collected from the solar salt works in Thamaraikulam, Kanyakumari district, Tamilnadu, India (Lat. 8° 11’ N and Long.77° 29’ E). Samples were collected in sterile polythene bags, trans-ported to the laboratory aseptically and stored at 4°C for further use. Water samples were serially diluted from 10^-1^ to 10^-8^ in sterile salt pan water and 100 μl of each dilution was spread onto sterile nutrient agar plates containing 5 to 20% NaCl. The plates were incubated at 37°C for 7 days. After incubation morphologically different colonies were identified by morphology and biochemical confirmations as well as based on the characteristics described in Bergey’s Manual of Systematic Bacteriology (Holt et al. 1994).

Genomic DNA (100 ng) isolated from halophilic Halomonas sp BS4 strain was amplified by PCR using 16 S rRNA universal primers (Forward: 5^′^ CAGGCCTAACACATGCAAGTC 3^′^; Reverse: 5^′^ GGGCGGWGTGTACAAGGC 3^′^). The PCR product was cloned into the vector pTZ57R and used to transform *Escherichia coli* DH5α as described by Sambrook et al.^1989^). The transformants were sequenced using an ABI 3700 automated DNA sequencer. Sequences were compared with other 16 S rRNAs obtained from GenBank using the BLAST program. The phylogenetic tree was constructed by MEGA5 software and evolutionary history was inferred using the UPGMA method (Sneath and Sokal, 1973). The evolutionary distances were computed using the Maximum Composite Likelihood method (Tamura et al. 2004) and are in the units of the number of base substitutions per site. The optimal tree with the sum of branch length = 0.31662628 is shown.

### Growth optimization of Halomonas sp BS4

Bacterial isolates were evaluated at various pH (5-10) and sodium chloride concentrations (2-20%) in nutrient broth to find out the optimum growth conditions. The optical density at 600 nm wavelength was measured for evaluating bacterial growth in broth culture.

### Biosurfactant screening, extraction and purification

Different biosurfactant screening methods were done for finding out potential biosurfactant producing halophilic Halomonas sp BS4. The methods adopted were (a) drop-collapse test by adding mineral oil in 96-well microtitre plates (Jain et al. 1991); (b) Oil spreading technique by adding weathered crude oil (Youssef et al. 2004); (c) Emulsification activity by adding kerosene and equal volume of cell free supernatant (Cooper and Goldenberg, 1987) and (d) Hemolytic activity in 5% blood agar plate.

The biosurfactant was extracted from cell-free broth at 72-h grown cells by step-by-step purification of acid precipitates using adsorption chromatography. Bacterial cells were removed from surfactant-containing medium by centrifugation (10,000 rpm for 20 min). The supernatant was subjected to acid precipitation by adding 6 N HCl to achieve a final pH of 2.0 and allowing precipitating at 4°C. The precipitate was pelleted at 10,000 rpm for 20 min, re-dissolved in distilled water, adjusted to pH 7.0, freeze-dried, and weighed. The dried surfactant was extracted with acetone and dried with the aid of a rotary evaporator under vacuum (Pruthi and Cameotra, 1997).

### Structural characterization of biosurfactants

The dried biosurfactants obtained from acid precipitation method was dissolved with distilled water and spotted on TLC (Merck) sheets and run with CHCl_3_/CH_3_OH/H_2_O (65:15:1) as mobile phase. The chromatogram was developed under short UV light as well as exposing iodine vapour. The R_f_ value was calculated as per the standard database of biosurfactants (Janek et al. 2010).

The basic functional groups of the purified biosurfactants from halophilic Halomonas sp BS4 were analyzed qualitatively by Fourier Transform Infra Red (FTIR) method described by Kemp (1991).

GC-MS analysis of partially purified biosurfactants were analysed individually using Agilent GC-MS 5975 Inert XL MSD (United States) gas chromatography equipped with J and W 122 – 5532G DB-5 ms 30 × 0.25 mm × 0.25 μm and mass detector (EM with replaceable horn) was operated in EMV mode. Helium was used as carrier gas with the flow rate of 1.0 ml min^-1^. The injection port temperature was operated at 250°C. The column oven temperature was held at 80°C for 2 min then programmed at 10°C min^-1^ to 250°C, which was held for 0 min, and then at 5°C min^-1^ to 280°C which was held for 9 min. Electron impact spectra in positive ionization mode were acquired between m/z 40 and 450.

### Pharmacological screening of biosurfactants

*In vitro* antibacterial activity was performed by the partial purified biosurfactants against few human pathogens (*Staphylococcus aureus, Klebsiella pneumonia, Streptococcus pyrogenes* and *Salmonella typhi*) using agar diffusion method and agar over layer method. The pathogens was inoculated at the rate of 1 × 10^-4^ cfu/ml. For anti fungal activity, 20 μl of biosurfactant was poured into a well made in the centre of Potato Dextrose Agar (PDA) plates and fungal spores (approximately 10 spores) were inoculated onto the plates and incubated at 35°C for 48 hr. The zone of inhibition was recorded.

Antiviral activity was performed against White Spot Syndrome Virus (WSSV) following the method of Balasubramanian et al. (2006). Five micro litre of purified WSSV suspension (300 μg of total protein) was mixed with different concentraions of biosurfactants (2, 4, 6, 8 and 10 μg/μl) and incubated at 29°C for 3 h. After incubation period, the mixture was injected intramuscularly to *Fenneropenaeus indicus* had the average weight of 10 ± 1 g. Three replicates were (n= 10 × 3= 30) maintained in all treatments. Mortalities were recorded daily and the experiment was carried out up to 10 days. Control shrimps were injected with a mixture of 10 μl NTE buffer and 5 μl viral suspensions. Haemolymph samples were collected from all injected shrimps and checked by WSSV diagnostic PCR using VP28 primer designed by Namita et al. (2007). The DNA extraction and PCR amplification were carried out by following the method described by Chang et al. (1999). Haemolymph samples of experimental and control shrimps were tested by the first step PCR. The negative samples detected in the first step were further subjected for second step PCR analysis. For this experiment, ethical clearance was obtained from Manonmaniam Sundaranar University ethical committee, Tirunelveli, India (Ref. : MSU/Ethical /2011/4 dtd 28.8.2011).

The anticancer activity was performed in tumor mammary epithelial carcinoma cell lines with the partial purified biosurfactants extracted from halophilic Halomonas sp BS4 following the method of Freshney (2007) and the activity was monitored after 48 hrs.

## Results

**Figure 1:**
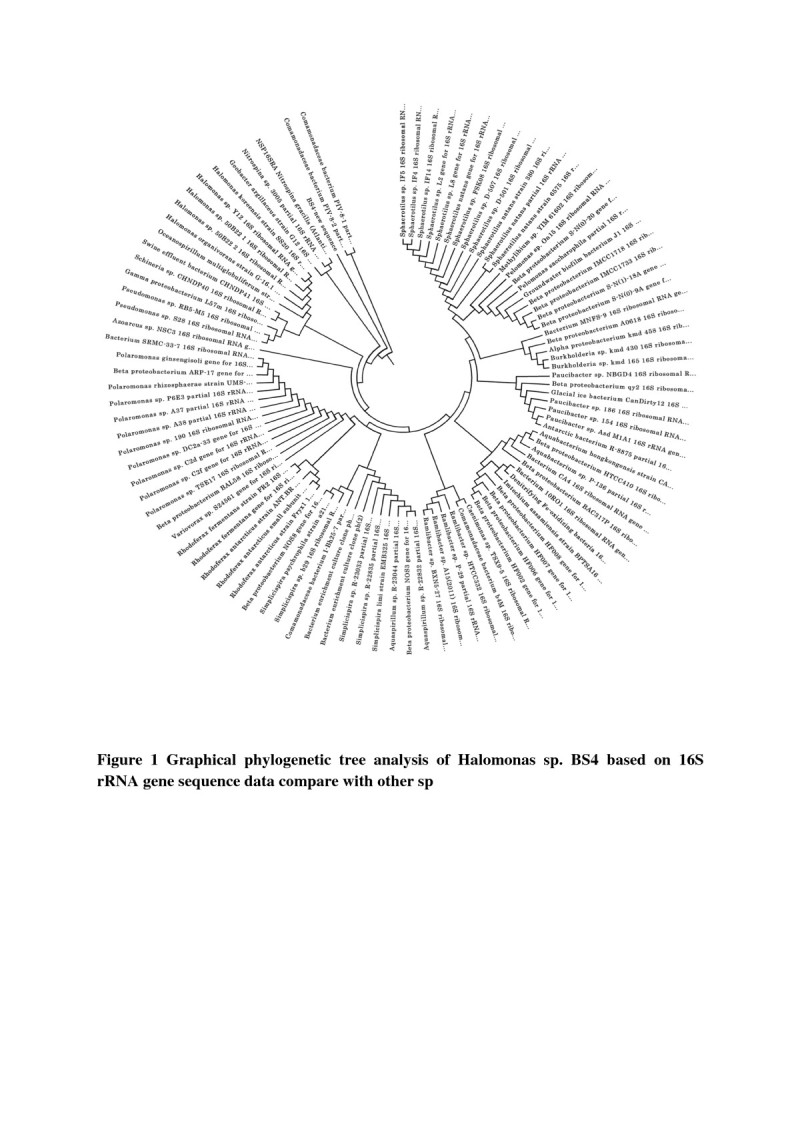
**Graphical phylogenetic tree analysis of Halomonas sp. BS4 based on 16S rRNA gene sequence data compare with other sp.**

Three different colonies were isolated from agar plates based on the colour such as pink, creamy and creamy white. The isolates were moderately halophilic (5-15%) and rod shaped. As per the biochemical results, the selected strains may be Halomonas sp BS4, Bacillus sp and *Bacillus subtilis* (Table 1). The selected bacterial strain (Halomonas sp BS4) was identified taxonomically using 16 S rRNA gene sequence. To determine their phylogenetic position, the 16 S rRNA gene sequence was analysed and phylogenetic tree was constructed. Phylogenetic analysis also indicated that the selected isolate BS4 belonged to the genera Halomonas (Figure 1).

**Table 1:** **Phenotypic identification of biosurfactant producing Halomonas sp BS4 isolated from solar salt works in India in comparison with other Halomonas sp**

Characteristics	Halomonas sp-BS4	***H. salina#***	***H. halophila#***	***H. elongate#***	***H. eurihalina#***
Colony Colour	Pink	Cream-yellow	Cream	Cream-beige	Cream
Cell morphology	Short rod	Short rod	Rod	Long Rod	Short Rod
Motility	+	ND	ND	ND	ND
Indole	-	ND	ND	ND	ND
Methyl Red	-	ND	ND	ND	ND
VP	-	ND	ND	ND	ND
NaCl range (% W/v)	0-20	2–20	2–30	0–25	3.5–25
NaCl optimum (% W/v)	8.0	5.0	7.5	11.0	2.0
pH range	5.0-10.0	5.0–10.0	5.0–10.0	5.0–9.0	5.0–10.0
Temperature range	15-45	4–45	10–45	15–45	4–45
Nitrate reduction	+	+	+	+	+
Oxidase	-	+	+	-	-
**Hydrolysis:**					
1. Gelatin	+	-	-	+	+
2. Urea	+	+	+	+	+
3. Tyrosine	+	+	-	-	+
EPS production	+	+	-	-	+
**Acid from:**					
1. D-Glucose	+	-	+	+	-
2. L- Arabinose	-	-	+	-	+
3. D-Galactose	-	-	+	-	-
4. Lactose	-	-	-	+	-
5. Maltose	+	-	+	+	-
6. Mannose	-	-	+	-	-
7. Sucrose	+	-	-	+	-
8. Trehalose	ND	-	+	+	-
GC Content (%)	53	60.5	59.1	60.4	66.7

#: The data obtained from Romano et al. (1997); *ND*: Not Determined.

**Figure 2:**
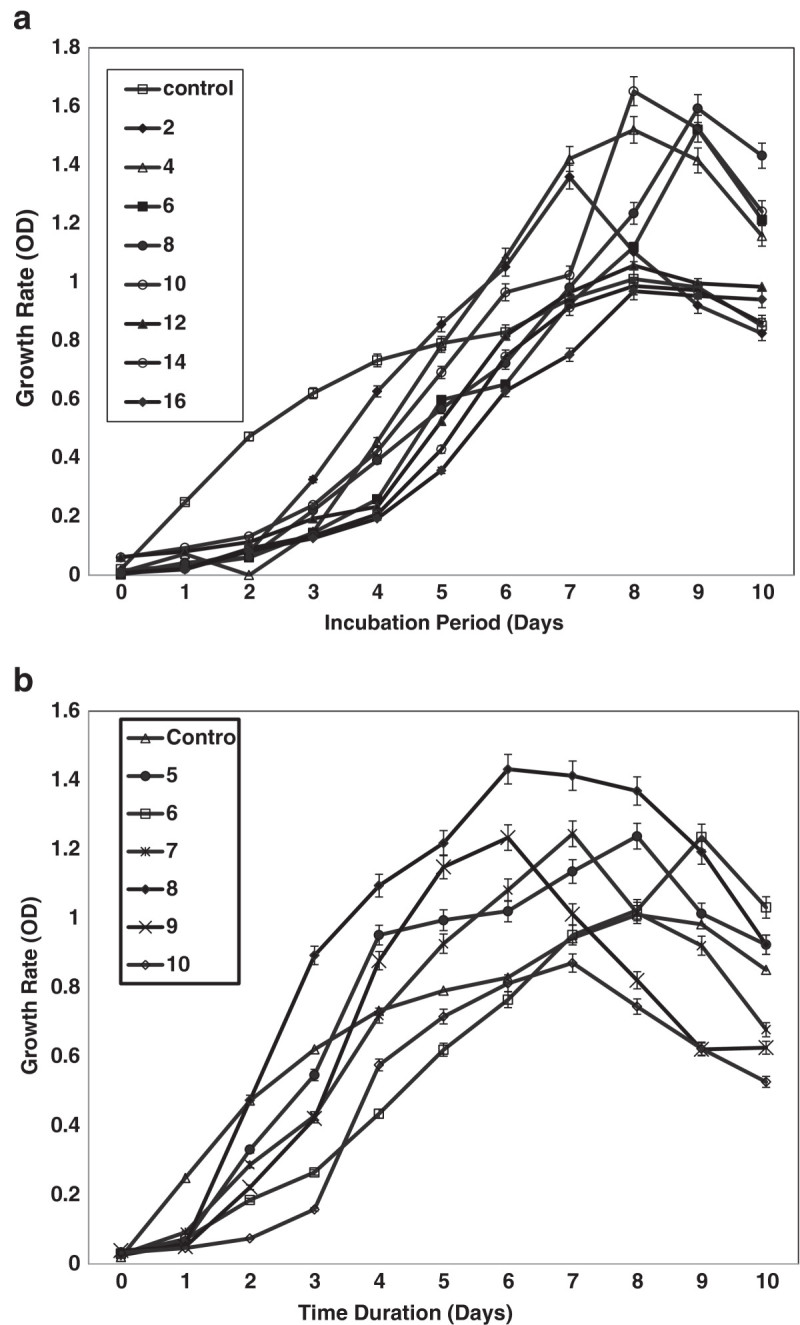
**Growth optimization of Halomonas sp BS4 isolated from solar salt works grown in NaCl concentrations (a) and pH (b).** The values are significantly differed each other’s (*F = 78.88;* P < = 0.001- Figure 2a) and (*F = 48.36;* P < = 0.001- Figure 2b) – Two Way ANOVA.

The growth condition of the selected strain (Halomonas sp BS4) was optimized by NaCl concentration and pH (Figure 2a and 2b). The purpose of optimization of the strains was to find their optimum growth. From the results it was concluded that the Halomonas sp BS4 grow highest on 8% NaCl. Similarly, pH was checked for the selected strain that grows best in the range of 6-8 pH.

Among the different biosurfactant screening tests such as drop collapse test, Oil spreading technique, Emulsification activity and Hemolytic activity, biosurfactant producing ability was found only in the selected strain Halomonas sp BS4 where as compared to the selected strain (Halomonas sp BS4) very low or negative result was observed from the other isolates (Bacillus sp and *Bacillus subtilis*) (Table 2).

**Table 2:** **Biosurfactant screening Halomonas sp BS4 isolated from solar salt works in India**

Bacterial strains	Drop collapse test	Oil spreading test	Emulsification activity	Haemolytic activity
Halomonas sp BS4*	**+++**	**+++**	**+++**	**++++**
Bacillus sp**	**-**	**-**	**-**	**-**
Bacillus subtilis***	**++**	**+**	**+**	**+**

* Halomonas sp BS4 isolated from solar salt works; ** Bacillus sp isolated from solar salt works and *** *Bacillus subtilis* isolated from back water of Rajakkamangalam, India.

**Figure 3:**
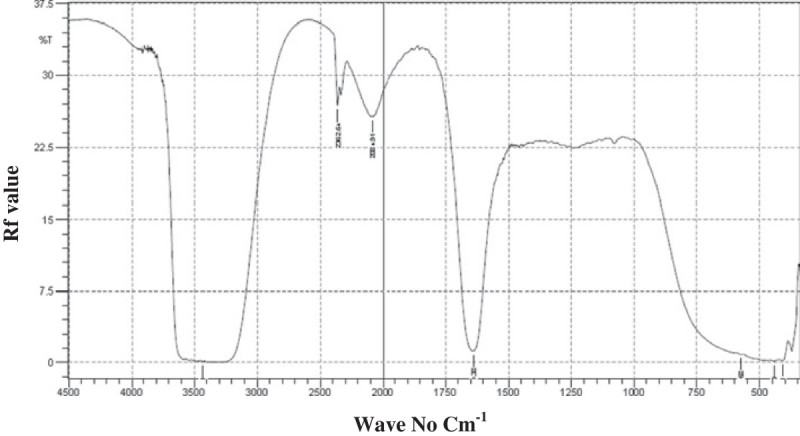
**Functional group analysis of Halomonas sp BS4 yield biosurfactants by Fourier Transmission Infra Red (FTIR) spectroscopic analysis.**

TLC analysis showed that the production of biosurfactant by Halomonas sp BS4 cultivated in mineral salt medium detected spots with Rf value of 0.40. The IR spectrum in KBr showed bands characteristic of peptides at 3431 cm^-1^ (NH stretching mode). The band at 2362 cm^-1^ is due to the presence cumulated system R_2_C = N = N in the sample. The absorption at 1639 cm^-1^ is possible due to either stretching of –C = C, stretching of carboxylate anion. The peak at 576 cm^-1^ and the peaks at 443 and 410 cm^-1^ confirm the presence of C-Br and C-I bond in the sample (Figure 3).

The fraction chosen for GC-MS gave peaks corresponding to long chain aliphatic compounds consistent with fatty acid methyl esters. It is highly unlikely that such compounds would exhibit a bioactive response when subjected to the tests performed in this study, indicating that the bioactive compound is located in one of the other fractions. The analyzed fraction was reasonably pure, with 3 main constituents, including 1, 2-Ethanediamine N, N, N’, N’-tetra,8-Methyl-6-nonenamide, (*Z*)-9-octadecenamide, the amide of oleic acid, which is a fatty acid derivative (Table 3).

**Table 3:** **Major compounds identified from the partial purified biosurfactants from Halomonas sp BS4 by GCMS analysis**

Sl. no	Retention time	Name of the compounds	Molecular formula	Molecular weight	Quality %
1.	2.133	1, 2-Ethanediamine, N, N, N’, N’-tetra	C_6_H_16_N_2_	116.2046	86
2.	30.333	8-Methyl-6-nonenamide	C_10_H_19_NO	169.2640	53
3.	30.411	9-Octadecenamide, (Z)	C_18_H_35_NO	281.4766	53

**Figure 4:**
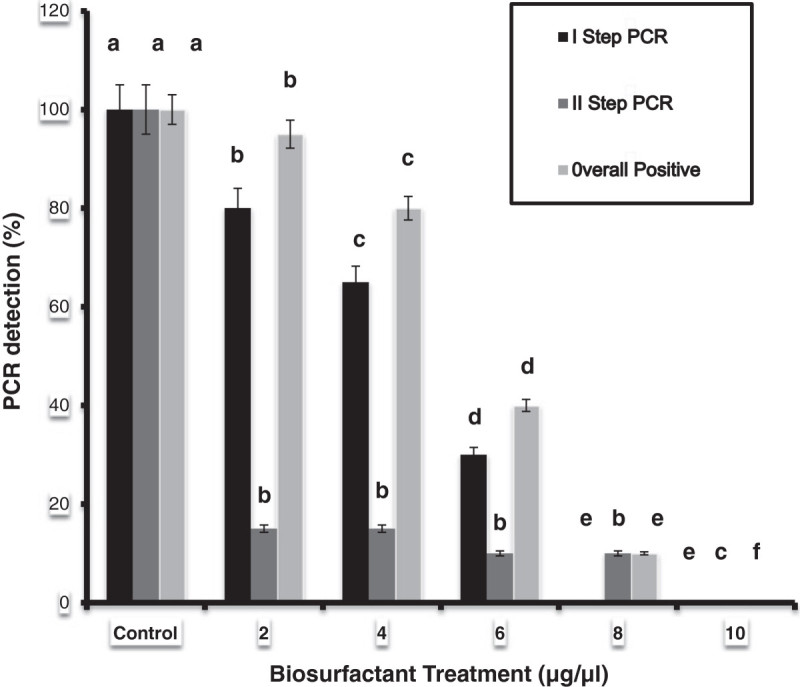
**PCR detection of haemolymph samples of*Penaeus monodon*after injection with various percentages of biosurfactant incubated with WSSV.** Statistical differences (P < 0.01) between treated and control groups are indicated by **a**-**f** superscripts; error bars are standard errors- One way ANOVA.

**Figure 5:**
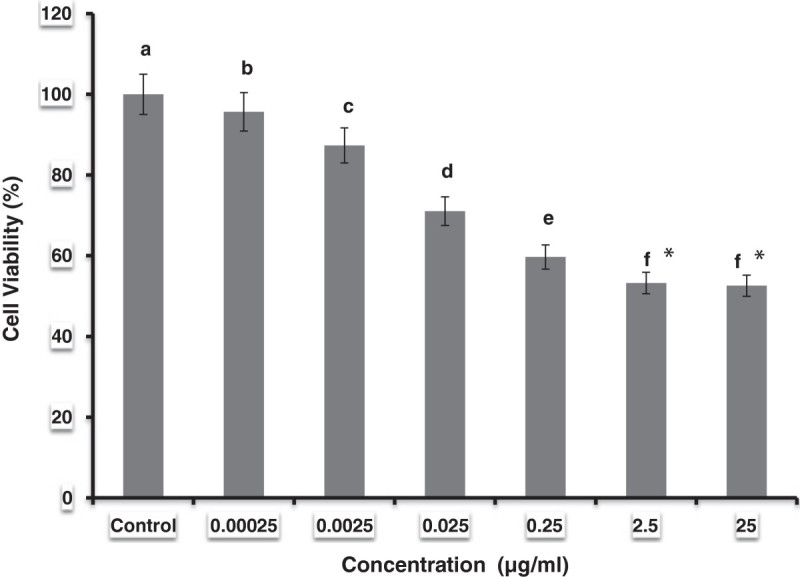
**Anti tumor activity performed in tumor mammary epithelial carcinoma cell lines with various percentages of Halomonas BS4 yield biosurfactants.** Statistical differences (P < 0.001) between treated and control groups are indicated by **a**-**f** superscripts and asterisks indicates non significant; error bars are standard errors- One way ANOVA.

The Halomonas sp BS4’s biosurfactants effectively inhibited the growth of pathogenic bacteria as well as fungi (Table 4). The antibacterial activity against *Staphylococcus aureus, Klebsiella pneumonia, Streptococcus pyrogenes* and *Salmonella typhi* was detected by observing a zone of inhibition of 15.35, 15.60, 11.98 and 17.33 mm respectively. A higher antifungal activity was observed against *Aspergillus niger* and *Fusarium* sp. while moderate activity was noticed against *Aspergillus flavus* and *Trichophyton rubrum*. The antiviral effect of the biosurfactant against White Spot Syndrome Virus (WSSV) revealed that, higher percentages (60, 80 and 100%) of biosurfactant effectively suppressed the growth/pathological effect of WSSV. All the WSSV injected shrimp in the positive control group succumbed to death within 5 days of post inoculation whereas the surfactant treated groups had prolonged survival rates and exhibited very less mortality of 70 and 90% at the end of experiment. One step PCR detection also supported the higher percentages of biosurfactants. There is no positive PCR signals observed in 80 and 100% of biosurfactants due to cent percent suppression of virus growth (Figure 4). Various concentrations of biosurfactants treated mammary epithelial carcinoma cell were given in the Figure 5. The concentrations of 0.00025 μg suppressed the cells of 4.44%; 0.0025 supressed 12.67%; 0.025 suppressed 28.95%, 0.25 suppressed 40.32% 2.5 suppressed 46.77% and 25 μg suppressed the maximum of 47.42%.

**Table 4:** ***In vitro*** **antibacterial and antifungal activity of partial purified biosurfactants from Halomonas sp BS4**

Sl. no	Antibacterial activity		Antifungal activity	
	Bacterial pathogens	Activity (mm of zone of inhibition)	Fungal pathogens	Activity
1	*Staphylococcus aureus*	15.35 ± 0.78^a^	*Trichophyton rubrum*	+++
2	*Klebsiella pneumonia*	15. 60 ± 0.85 ^a^	*Aspergillus niger*	++++
3	*Streptococcus pyrogens*	11.98 ± 0.45 ^b^	*Aspergillus flavus*	+++
4	*Salmonella typhi*	17.33 ± 0.15 ^c^	*Fusarium sp*	++++

++++: higher activity; ++: medium activity; +: less activity.

Means with the same superscripts (a-c) do not differ from each other (P < 0.001)- One way ANOVA.

## Discussion

Biosurfactants are produced by several types of microorganisms, such as bacteria, fungi and yeasts (Fiechter 1992). Some microorganisms are capable of growing in extreme environments, where most other organisms are not able to survive. Among these extremophiles, halophiles are one of the major microbial communities that tolerates high salt concentrations and are highly sought after by many industries for their novel enzymes and products that has wider potential applications (Ventosa et al. 1998). Halomonas is a Gram-negative bacterium, non-spore forming, predominantly present in marine environments and are often isolated from deep-sea sediments and hydrothermal vents. Halomonas sp. comprises a remarkably high percentage (up to N10%) of the total microbial community in their habitat (Kaye and Baross 2000). Most of the Halomonas sp. that has been reported for EPS production has been isolated from hypersaline environments with different salt concentration (Martínez-Checa et al. 2002; Quesada et al. 2004a). Gutierrez et al. (2009) isolated and studied physicochemical properties of EPS namely HMWEPS from Halomonas sp. strain TG39 by growing on different types of substrates.

There are very few reports on biosurfactant producers in hypersaline environments (Cameotra and Makkar 1998). Halophiles, which have a unique lipid composition (phytanylglycerol), may have an important role to play as surface-active agents. The archae bacterial ether-linked phytanyl membrane lipid of the extremely halophilic bacteria has been shown to have surfactant properties (Post and Collins 1982). According to Tsuge et al. (1996), lipopeptide surfactants are potent antibiotics mainly the surfactin, streptofactin and gramicidin produced by the microorganism had the wide antimicrobial activity (Peypoux et al. 1999). This is consistent with the fact that 50% of secondary metabolites are terpenes, 25% are acteogenins (polyketides) and 25% are fatty acid derived (Blackman 2005). As previously described for rhamnolipid molecules containing two different 3-hydroxy fatty acid side chains, rhamnolipid molecules with the shorter 3-hydroxy fatty acid side chain are found to be more abundant than those with the longer chain connected to the rhamnose molecule at the same position (De’ziel 2000).

The present study TLC analysis revealed that, the Rf value of 0.40 confirmed as Glycolipid. George and Jayachandran (2009), studied the production of biosurfactant from *P. aeruginosa* MTCC2297 cultivated in orange fruit peelings detected spots with Rf values of 0.19 (dirhamnolipids), 0.36 (monorhamnolipids), 0.59 and 0.71 (various rhamnolipid forms), 0.82 and 0.98 in silica plates. Haba et al. (2000), on the other hand, observed that Pseudomonas sp. cultivated in medium supplemented with used vegetable oils produced a mixture of two rhamnolipids with Rf value of 0.7 and 0.45. Arino et al. (1996) characterized the rhamnolipid mixture produced by *P*. *aeruginosa* GL1. The Rf values for different spots were calculated and it corresponds to R1 0.72 (Rha-C10C10,), R2 0.40 (Rha-C10), R3 0.32 (Rha-Rha-C10C10) and R4 0.13 (Rha-Rha- C10). The Glycolipid biosurfactant was characterized from Halomonas sp by FTIR and TLC analysis. The GC –MS analysis revealed that, the biosurfactant of halophilic Halomonas sp BS4 contain polymers, fatty acids and other compounds including 1, 2-Ethanediamine N, N, N’, N’-tetra, 8-Methyl-6-nonenamide, (*Z*)-9-octadecenamide etc. In the present study, the biosurfactant contain, 1, 2-Ethanediamine N, N, N’, N’-tetra, at the quality of 86% and 9-Octadecenamide, (Z) at 53% quality. The sponge associated actinomycetes, *Nocardiopsis dassonvillei* MAD08 contains 9-octadecenamide (Z) that had the broad range of antimicrobial activity including anticandid activity (Selvin et al. 2009). 1,2-Ethanediamine, N,N,N’,N’-tetramethyl- is a polymeric biosurfactant also present in the biosurfactant of the Halomonas sp BS4 at high quality level. 1,2-Ethanediamine, N,N,N’,N’-tetramethyl- had a broad pharmacological activities including anti tumor and antifungal activities. Its anti fouling activity in aqueous system is also shown to be microbicidal and thus preventing adhesion of bacteria. This polymeric biosurfactant also suppressed the motility, temporary attachment of hydroid larvae *Dynarnena pumila* and *Obelia loveni* and prevent the attachment and contractility of young blue mussels *Mytilus edulis* (Railkin^1994^). Halomonas species produce emulsifiers which is effective in a wide range of food oils under both neutral and acidic pH conditions, even under high temperature and acidic conditions (Gutierrez 2009).

The ability of a mixture of seven different rhamnolipids to inhibit microbial growth was determined by Abalos et al. (2001). Low concentrations were able to inhibit the bacteria *Staphylococcus epidermidis*, *E*. *coli* and *Alcaligenes faecalis*. Also inhibited the growth of fungi *Aspergillus niger*, *Glicadiam virans*, *Boryttis cinera* and *Penicillium crysogenum*. The present results revealed that the biosurfactants extracted from Halomonas sp BS4 were able to suppress the bacteria *Staphylococcus aureus, Klebsiella pneumonia, Streptococcus pyrogenes* and *Salmonella typhi* at more than 10 mm of zone of inhibition. Isoda et al. (1999) investigated the antibacterial activities of seven extracellular microbial glycolipids, including MEL-A, MEL-B, polyol lipid, rhamnolipid, SL and succinoyltrehalose lipids STL-1 and STL-3. The biosurfactant also effectively suppress the growth of the fungi such as *Aspergillus niger, Trichophyton rubrum,* Fusarium sp and *Aspergillus flavus.* Nielsen et al. (1999) reported viscosinamide, a cyclic depsipeptide, to be a new antifungal surface-active agent produced by *Pseudomonas fluorescens*, with different properties compared with the biosurfactant viscosin.

Glycolipids have also been implicated in growth arrest, apoptosis and the differentiation of mouse malignant melanoma cells (Zhao 2000). The biosurfactant also have some anticancer activities, they suppress the cell viability in tumor mammary epithelial carcinoma cells at 25% in the 25 μg concentrations. Arena et al. (2009 Vollenbroich et al.) reported novel type of EPS-1 polysaccharide had an antiviral and immunomodulatory effect which was produced by thermo tolerant strain *Bacillus licheniformis.* (1997) showed that surfactin is active against several viruses, including Semliki Forest virus, herpes simplex virus (HSV), Suid herpes virus, vesicular stomatitis virus, simian immunodeficiency virus, feline calicivirus and murine encephalomyocarditis virus. In the present study, the biosurfactant incubated WSSV injected shrimps had improved survival when compared with the control. The 100% biosurfactant treated group had increased survival to 3 times. The present findings revealed that the biosurfactant isolated from the Halomonas sp BS4 had wider pharmacological activities and this will help to develop novel drugs. Further studies are needed to improve the biosurfactant production and purification of the various compounds such as glycolipids, polymeric substances and lipopeptides from the Halomonas sp BS4.

## Authors’ original submitted files for images

Below are the links to the authors’ original submitted files for images. Authors’ original file for figure 1 Authors’ original file for figure 2 Authors’ original file for figure 3 Authors’ original file for figure 4 Authors’ original file for figure 5
