# Supplementary figures and images for: Halomonas sp. BS4, A biosurfactant producing halophilic bacterium isolated from solar salt works in India and their biomedical importance
Source: Springerplus. 2013 Apr 10;2(1):149. doi: 10.1186/2193-1801-2-149 (PMC3648683; doi:10.1186/2193-1801-2-149)

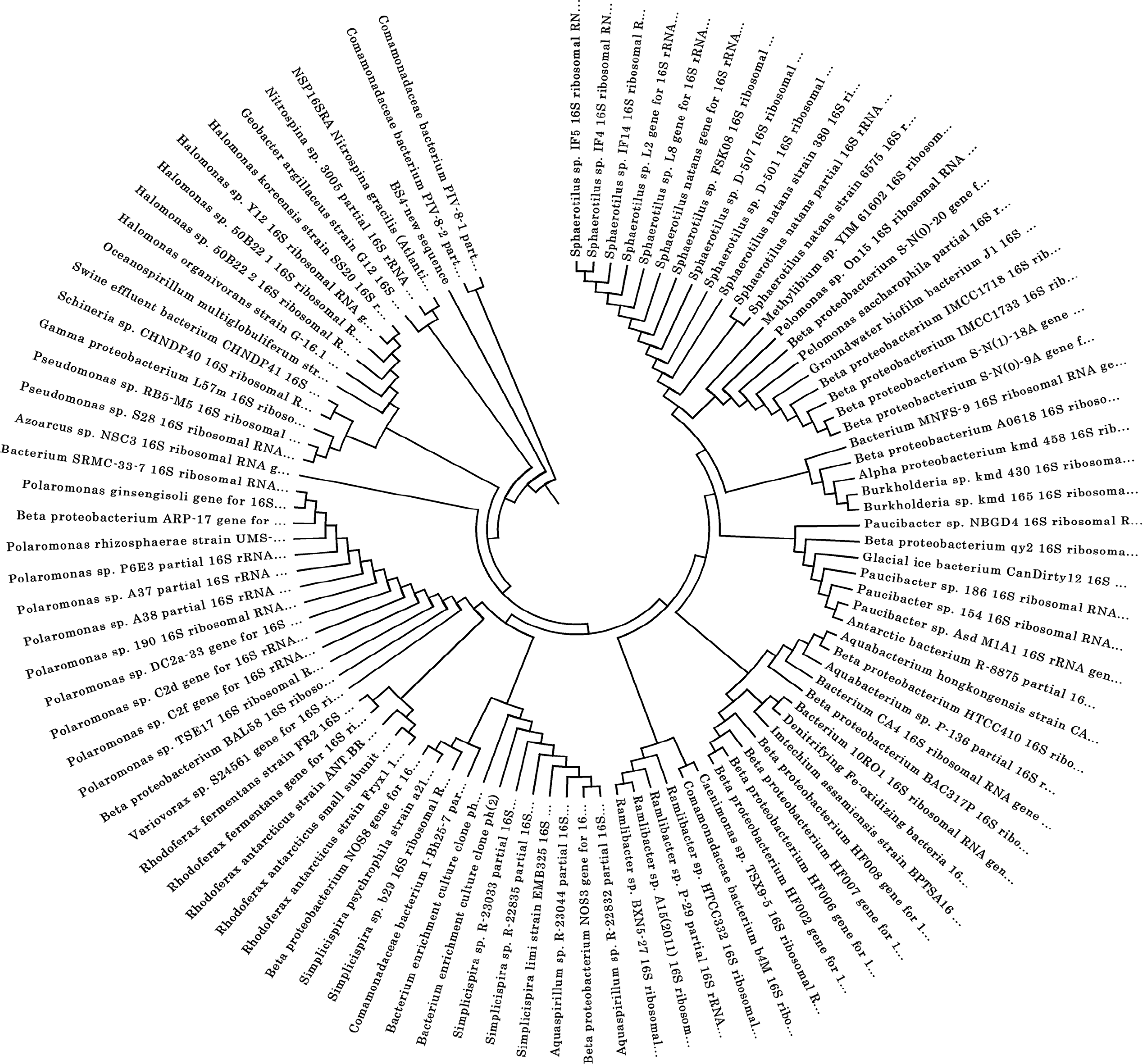

Supplement: Supplementary file 1 — Authors’ original file for figure 1 [file 40064_2013_244_MOESM1_ESM.tiff]

**a**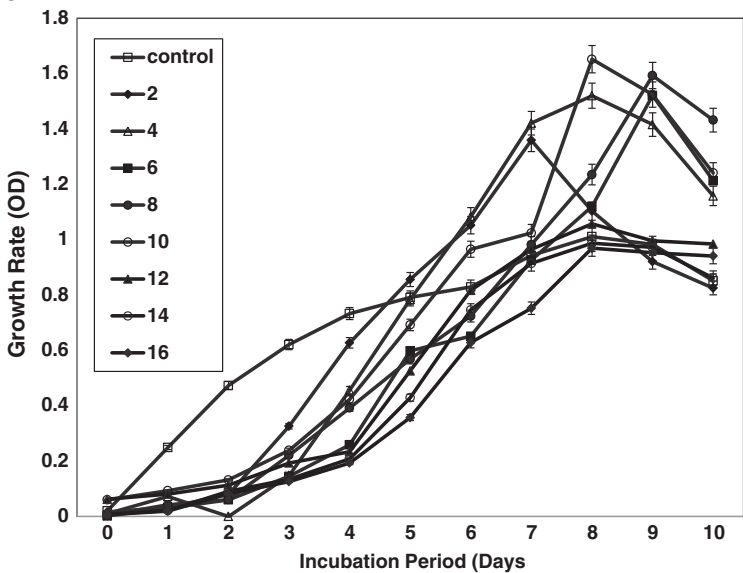**b**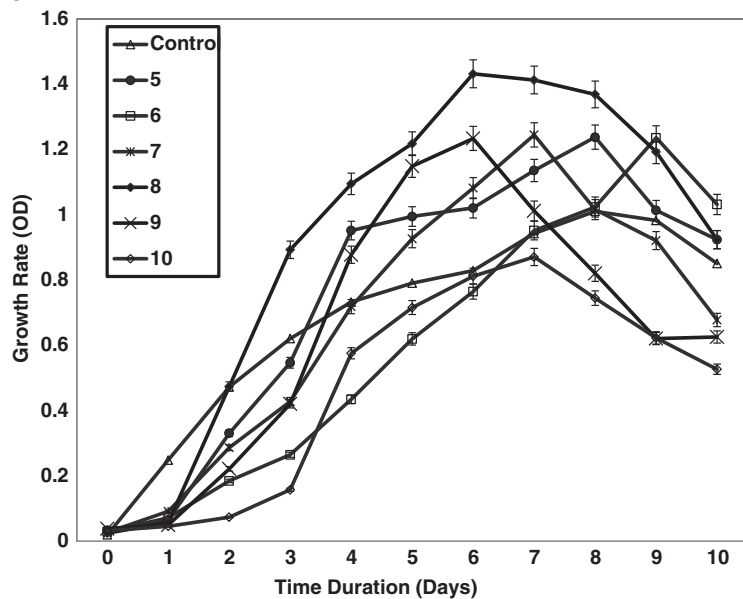

Supplement: Supplementary file 2 — Authors’ original file for figure 2 [file 40064_2013_244_MOESM2_ESM.pdf]

Rf value

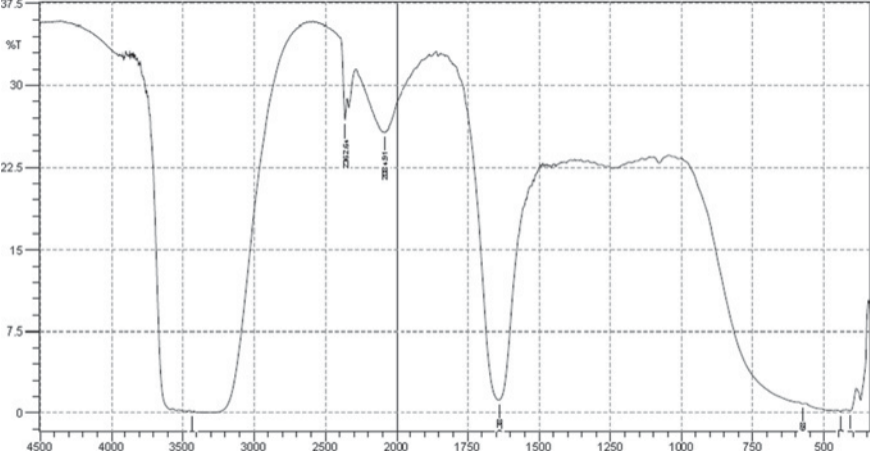

Wave No  $\text{Cm}^{-1}$

Supplement: Supplementary file 3 — Authors’ original file for figure 3 [file 40064_2013_244_MOESM3_ESM.pdf]

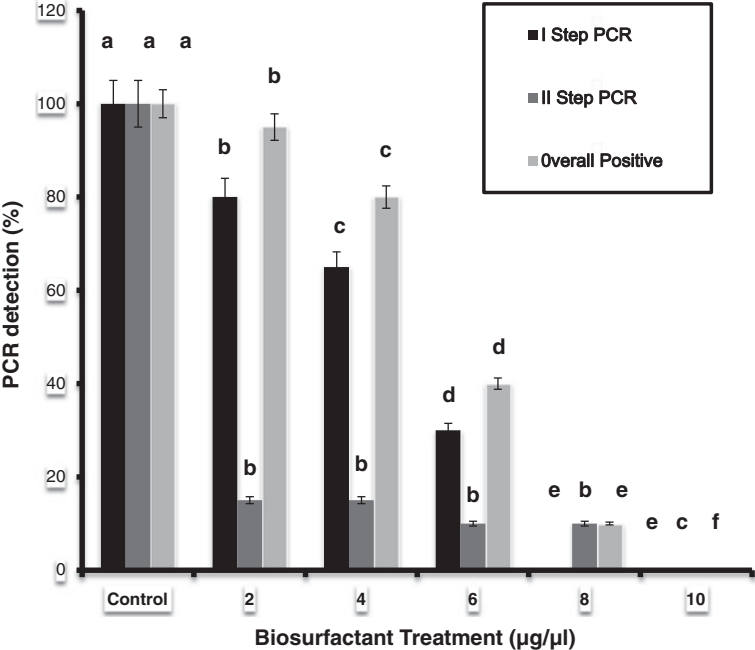

Supplement: Supplementary file 4 — Authors’ original file for figure 4 [file 40064_2013_244_MOESM4_ESM.pdf]

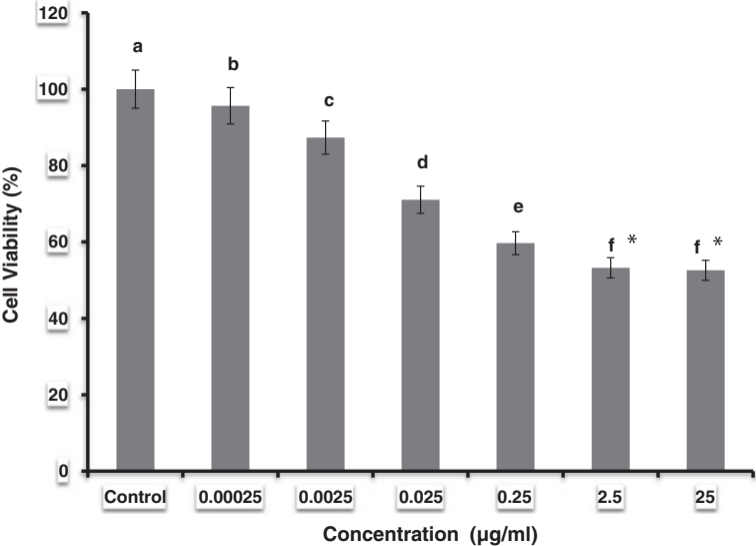

Supplement: Supplementary file 5 — Authors’ original file for figure 5 [file 40064_2013_244_MOESM5_ESM.pdf]
